# Fluorescent Sensor Array Based on Black Plum Peels-Derived Carbon Dots for Multiplex Heavy Metal Ions Identification

**DOI:** 10.3390/bios16070372

**Published:** 2026-07-08

**Authors:** Ling Yang, Dandan Peng, Haihu Tan, Yahu Wang, Xin Lu, Fanming Zeng, Shi Gang Liu, Yuejun Liu

**Affiliations:** 1 School of Packaging Engineering, Hunan University of Technology, Zhuzhou 412007, China; yangling2020@hut.edu.cn (L.Y.);; 2 National & Local Joint Engineering Research Center for Advanced Packaging Material and Technology, Hunan University of Technology, Zhuzhou 412007, China; 3 College of Materials Science and Engineering, Fuzhou University, Fuzhou 350108, China; 4 Hunan Provincial Key Laboratory of Food Science and Biotechnology, College of Food Science and Technology, Hunan Agricultural University, Changsha 410128, China

**Keywords:** fluorescent sensor array, carbon dots, Heavy metal ions, LDA, HCA

## Abstract

Accurate discrimination of multiple heavy metal ions is essential for environmental monitoring. This study developed a simple fluorescent sensing array utilizing carbon dots derived from black plum peels (PCDs) for the precise identification of metal ions in environmental waters. Three structurally distinct PCDs were hydrothermally synthesized using phenylenediamine isomers as nitrogen dopants, exhibiting distinct fluorescence response patterns to target ions. Pattern recognition was performed using linear discriminant analysis (LDA) and hierarchical clustering analysis (HCA). The optimized system (pH 5–7) achieved high discrimination accuracy for eight metal ions (Sn^2+^, Ag^+^, Hg^2+^, Fe^3+^, Cr^3+^, Pb^2+^, Sb^3+^, and Cu^2+^) at 5–400 μM concentrations. The array effectively identified the binary and ternary mixtures of Hg^2+^/Cu^2+^/Cr^3+^ and successfully detected target ions in river water samples. This cost-effective and scalable approach demonstrates strong potential for applications in water quality monitoring and food safety.

## 1. Introduction

The exponential growth of industrialization and urbanization have intensified heavy metal pollution in aquatic systems. Notably, persistent heavy metals including lead (Pb), mercury (Hg), and chromium (Cr) migrate into the food chain via water–soil pathways, ultimately inducing neurotoxicity and carcinogenicity [1,2]. While trace levels of certain metal ions (e.g., Cu^2+^ and Fe^3+^) serve as essential micronutrients, their excessive accumulation can lead to toxicity effects [3,4]. Sensitive detection and accurate identification of heavy metal ions therefore demands advanced analytical methodologies.

In recent years, spectroscopic analytical techniques including atomic absorption spectrometry (AAS), atomic emission spectrometry (AES), atomic fluorescence spectrometry (AFS), and inductively coupled plasma mass spectrometry (ICP-MS) have dominated in the detection of metal ions. While offering exceptional sensitivity and analytical reliability, these methods are constrained by prohibitive instrumentation costs, cumbersome sample processes, and specialized operation requirements and thus face application bottlenecks in resource-constrained scenarios [5,6,7]. In contrast, fluorescence probe technology serves as a cost-effective, high-throughput, highly sensitive detection platform with minimal sample damage, capable of generating visual signal outputs, and featuring simple instrument operation [8,9,10]; it also facilitates the development of integrated sensors for the Internet of Things [11].

Compared with conventional fluorescent probes (e.g., organic small molecules [12], polymeric molecules [13], metal nanoclusters [14], semiconductor quantum dots [15]), carbon dots (CDs) exhibit distinct advantages as emerging fluorescent nanomaterials, including enhanced photostability, tunable emission, superior water solubility, excellent biocompatibility, simple surface functionalization, and scalable low-cost synthesis. These properties have garnered extensive attention for CDs in chemical and biosensing fields [16,17,18]. Notably, CDs have recently emerged as a versatile fluorescent probe platform in heavy metal ions detection. Pablo et al. synthesized sulfur-doped carbon dots (S-C-dots) via a one-step base-catalyzed method under ambient conditions using chicken egg white and L-cysteine, enabling Cu^2+^ sensing with a detection limit of 52 nM [19]. Jaison et al. developed yellow-emitting CDs from 5-dimethylaminomethylfurfuryl alcohol and *o*-phenylenediamine, achieving Hg^2+^-selective detection (limit of detection (LOD): 5.2 nM; linear range: 15–100 µM) [20]. Singh et al. fabricated blue-fluorescent CQDs from citric acid/ethylenediamine, demonstrating dual sensing of Fe^3+^/Hg^2+^ (LODs: 0.406 μM and 0.934 μM, respectively) within 0–50 μM while maintaining exceptional photostability [21]. Huang et al. engineered a reversible fluorescent switch by incorporating N-doped carbon dots (N-CDs) into a polyacrylamide-based hydrogel, allowing simultaneous Cu^2+^ (LOD: 0.813 μM) and Ag^+^ (LOD: 0.468 μM) recognition [22]. Despite these advances, current systems remain confined to single/dual-ion detection due to cross-interference and selectivity limitations in complex matrices.

Fluorescence sensing arrays, termed the “electronic tongue/nose”, address these challenges by generating differential response fingerprints for target recognition [3,23]. CDs-based arrays show particular promise in metal ions discrimination. Fu et al. synthesized bone-derived carbon dots through a hydrothermal method combining high-temperature pyrolysis with strong acid oxidation, constructing a multichannel array that specifically identified Ag^+^, Cu^2+^, Hg^2+^, Fe^3+^, Pb^2+^ and their binary/ternary mixtures, which achieving synchronous polymetallic detection in environmental water samples [24]. Qin et al. engineered a six-component sensing system through self-assembly of CDs with five amino acids, attaining 100% classification accuracy for 11 metal ions via fluorescence pattern recognition [3]. Xu et al. integrated CDs with EDTA-Tb^3+^ complexes into a multi-emission array, which achieved 95.6% discrimination accuracy for seven heavy metal ions (0.05–50 μM) assisted by machine learning, with 93.3% (lake water) and 100% (soil) accuracy in real-sample validation [25]. Nevertheless, these arrays are hindered by distinct defects in sensing units, including complex fabrication processes, high material costs, uncontrollable performance optimization, and limited analyte diversity.

Most reported CD-based fluorescent arrays rely on conventional chemical raw materials or common biomass precursors. Black plum peel, a widespread agricultural byproduct, is rich in cellulose, polyphenols, hydroxyl and carboxyl groups. It is low-cost, easily collected and conforms to the green synthesis concept [26]. After hydrothermal carbonization, it can provide abundant surface active sites for CDs, and the obtained CDs present diversified fluorescence responses to different heavy metal ions, which is ideal for constructing cross-reactive sensing arrays. To fill these research gaps, this work selected black plum peel as the carbon source and three phenylenediamine isomers as nitrogen dopants to prepare structurally differentiated CDs via a simple one-step hydrothermal method.

**Scheme 1:**
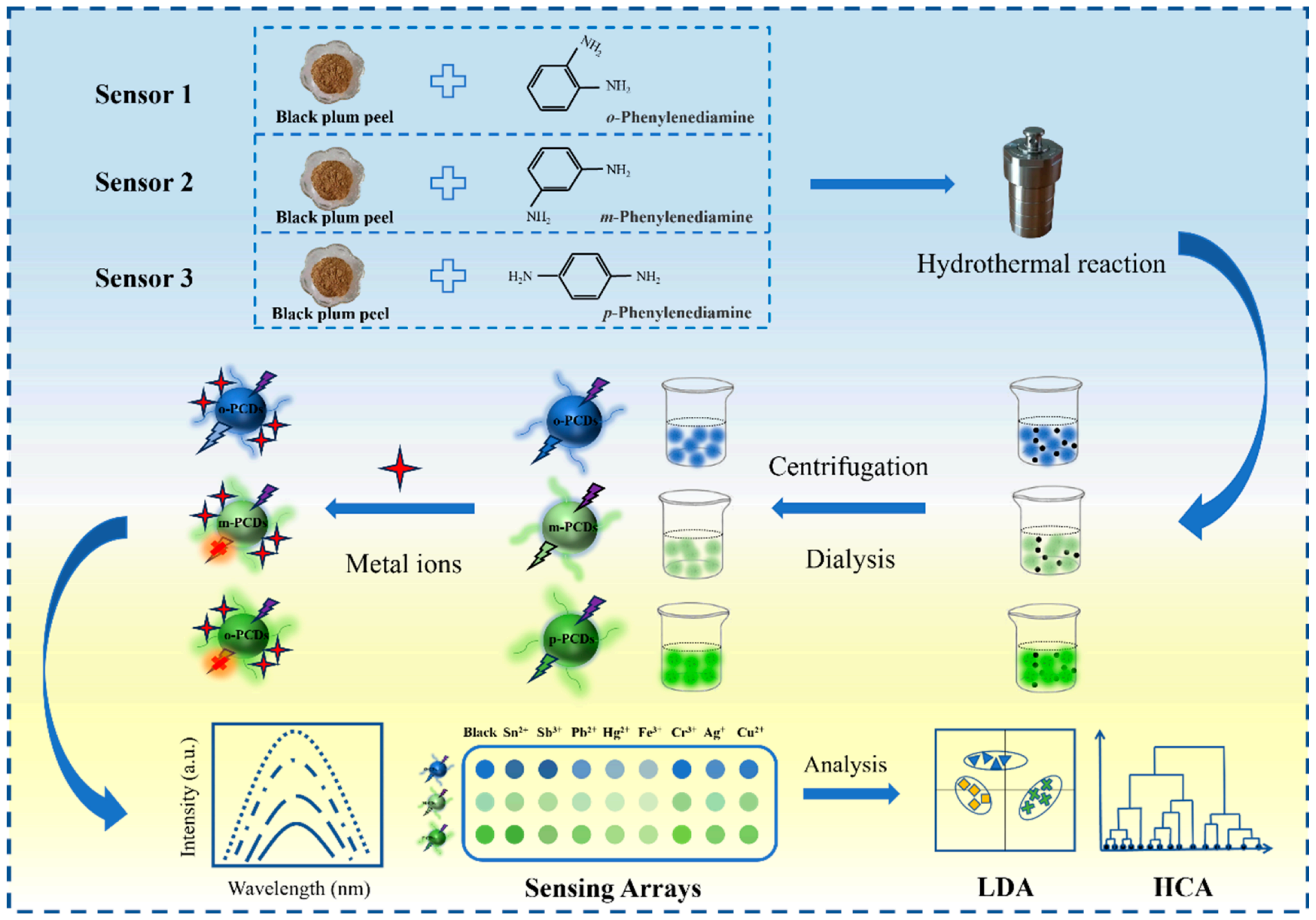
The fluorescent sensor array based on PCDs for the identification and discrimination of eight heavy metal ions.

Given the significant influence of nitrogen doping on the optical properties of CDs [27], this work synthesized three structurally controllable carbon dots derived from black plum peels (PCDs) via nitrogen-source modulation. Exploiting differential fluorescence responses induced from coordination interactions between PCDs with metal ions, a multichannel sensing array was engineered. Integrated with linear discriminant analysis (LDA) and hierarchical cluster analysis (HCA), the platform achieved precise discrimination of individual metal ions (Sn^2+^, Ag^+^, Hg^2+^, Fe^3+^, Cr^3+^, Pb^2+^, Sb^3+^, and Cu^2+^) and their binary/multicomponent mixtures. Optimized protocols enabled rapid identification in environmental water samples, validating environmental adaptability (Scheme 1). The fluorescence pattern-based strategy establishes a robust platform for simultaneous monitoring of heavy metal contaminants in complex matrices.

## 2. Experimental

### 2.1. Materials

Black plum was procured from a local supermarket (Hunan, China). Metal salts (SnCl_2_, HgSO_4_, FeCl_3_, PbCl_2_, CuCl_2_, SbCl_3_, and CrCl_3_·6H_2_O), phenylenediamine isomers (*o*-, *m*-, *p*-), NaCl, NaH_2_PO_4_, Na_2_HPO_4_, Na_2_CO_3_, NaF, Na_2_SO_4_, NaNO_3_, and NaOH were purchased from Aladdin (Shanghai, China). HCl and AgNO_3_ were sourced from Zhuzhou Quartz Glass Co., Ltd. (Zhuzhou, China) and Shanghai Shenbo Chemical Co., Ltd. (Shanghai, China), respectively. All reagents were of analytical grade and used without further purification. Aqueous solutions were prepared with deionized water. The environmental water samples were collected from the Xiangjiang River (Hunan, China).

### 2.2. Instruments

Fluorescence spectra were acquired using a fluorescence spectrophotometer (Hitachi F-4500, Tokyo, Japan), and fluorescence lifetime was measured with a steady-state/transient fluorescence spectrometer (Hitachi F7100, Tokyo, Japan). Fourier-transform infrared (FT-IR) spectra were obtained via a Nicolet 380 spectrometer (Thermo Scientific, Waltham, MA, USA). Ultraviolet–visible (UV-vis) absorption spectra were recorded on a TU-1810 spectrophotometer (Beijing Purkinje General Instrument Co., Ltd., Beijing, China). X-ray photoelectron spectroscopy (XPS, PHI 5000 Versaprobe III, Chigasaki, Japan) was employed to analyze the elemental composition and chemical structure of the PCDs surfaces. Field-emission transmission electron microscopy (JEOL JEM-F200, Tokyo, Japan) was utilized to observe the microscopic morphology and lattice fringes of the PCDs. The potential of PCDs was analyzed using a Zeta potential analyzer (Zeta, Nanolink SZ902M, Zhuhai Truth Optical Instruments Co., Ltd., Zhuhai, China).

### 2.3. Synthesis of PCDs

PCDs were synthesized via a hydrothermal method. Dried black plum peels were ground into powder, and 0.2 g of the powder was mixed with 0.1 g of *o*-, *m*-, or *p*-phenylenediamine isomer in 10 mL deionized water. The mixture was ultrasonicated for 3 min, transferred to an autoclave, and subjected to hydrothermal treatment at 190 °C for 5 h. After cooling to room temperature, the product was filtered through a 0.22 μm aqueous-phase membrane to remove aggregates and dialyzed (MWCO: 1000, 48 h) to eliminate residual precursors. The purified PCDs, denoted as *o*-PCDs, *m*-PCDs and *p*-PCDs based on the isomers used, were stored at 4 °C.

### 2.4. Metal Ions Detection Based on the PCDs Sensor Array

Then, 0.2 mL aliquots of the purified PCDs solution were diluted to 100 ML with deionized water. Equal volumes of the diluted solutions were mixed with the target ion (Sn^2+^, Ag^+^, Hg^2+^, Fe^3+^, Cr^3+^, Pb^2+^, Sb^3+^, and Cu^2+^) solutions at gradient concentrations (5–400 Mm), respectively. After 1 min incubation at room temperature, fluorescence intensity (I) was measured at 365 nm excitation. A unified excitation wavelength of 365 nm was selected in this work, which matches the wavelength of commercial portable UV lamps and facilitates the development of on-site detection devices. Although this wavelength is not the optimal excitation wavelength for individual PCDs, it can still maintain distinct differential fluorescence responses for metal ions without reducing discrimination performance. The fluorescence response ((I − I_0_)/I_0_) was calculated relative to the ion-free control (I_0_). Array reliability was validated through quintuplicate trials (3 sensor units × 8 ions × 5 replicates × 6 concentrations). Multidimensional fluorescence data were subjected to dimensionality reduction in SPSS (Statistics 26.0), followed by LDA and HCA to construct classification models for performance evaluation. Cross-validation, confusion matrix analysis and blind sample tests were further conducted to verify the robustness of classification models.

Binary (Cu^2+^/Hg^2+^, Cu^2+^/Cr^3+^, Cr^3+^/Hg^2+^) and ternary (Hg^2+^/Cr^3+^/Cu^2+^) ion mixtures were prepared at a total concentration of 100 μM and analyzed following the single-ion protocol.

### 2.5. Analysis of the Real Samples

To further assess the universality of the sensor array, filtered river water (0.22 μm pore size) was mixed with the target ions (100 μM) and was analyzed identically.

## 3. Results and Discussion

### 3.1. Synthesis and Characterization of PCDs

**Figure 1:**
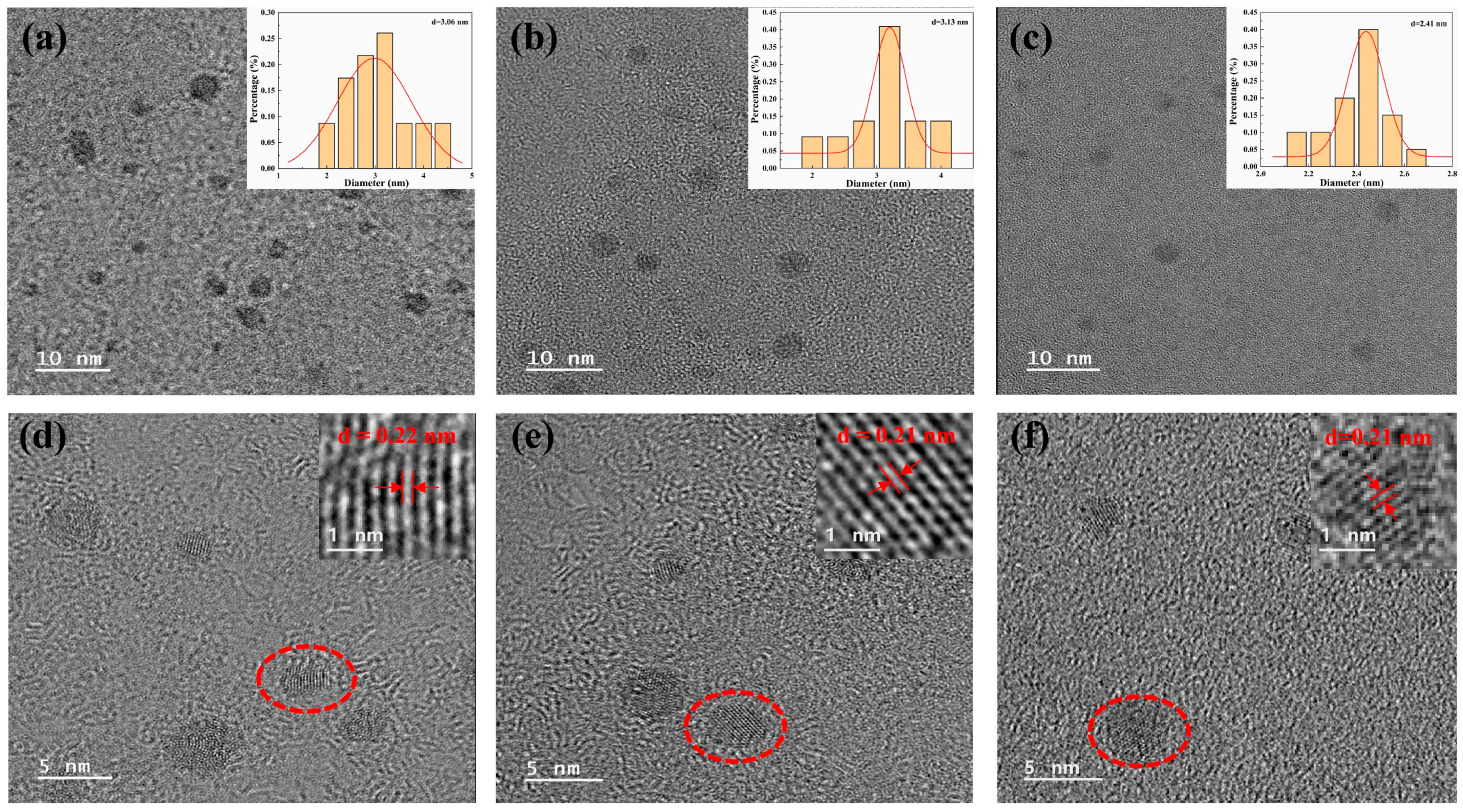
TEM (inset: particle size distribution) and HRTEM of (**a**,**d**) *o*-PCDs, (**b**,**e**) *m*-PCDs, and (**c**,**f**) *p*-PCDs.

Three PCDs, designated *o*-PCDs, *m*-PCDs, and *p*-PCDs, were hydrothermally synthesized using black plum peels and *o*-, *m*-, or *p*-phenylenediamine as respective precursors. Transmission electron microscopy (TEM) images (Figure 1a–c) reveal that all three types exhibit a quasi-spherical morphology, good dispersion, and narrow size distribution. Statistical analysis of over 100 particles yielded average diameters of 3.06 nm (*o*-PCDs), 3.13 nm (*m*-PCDs), and 2.41 nm (*p*-PCDs) (insets, Figure 1a–c). High-resolution TEM (HRTEM) images (Figure 1d–f) demonstrate well-resolved lattice structures with lattice spacings of 0.22 nm, 0.21 nm, and 0.21 nm, respectively, corresponding to the (100) plane of graphitic carbon [28].

The surface structures of *o*-PCDs, *m*-PCDs, and *p*-PCDs were characterized by FT-IR and XPS. Results indicate shared features alongside distinct differences.

FT-IR spectra (Figure S1) reveal surface functional groups. A broad peak at 3357 cm^−1^ is assigned to O-H/N-H stretching vibrations. Peaks at 2932 cm^−1^ and 880–680 cm^−1^ originate from alkyl C-H stretching vibrations and aromatic C-H out-of-plane bending vibrations, respectively. A characteristic peak near 1593 cm^−1^ corresponds to C=C/C=O/C=N stretching vibrations. The absorption peak at 1404 cm^−1^ is attributed to C-NH-C stretching vibrations, while the peak near 1080 cm^−1^ arises from C-N/C-O stretching vibrations. Spectral similarities among the samples, with only minor intensity and wavenumber variations, arose from their common precursors and synthesis method. The spectra confirm abundant surface hydrophilic groups (e.g., -COOH, -OH, and -NH_2_), which impart hydrophilicity and render the PCDs suitable as fluorescent probes [25,29].

The XPS analysis further elucidated the surface composition and structure of the three PCDs. The full spectrum of *o*-PCDs (Figure S2a) exhibits characteristic peaks derived from C 1s (285 eV), N 1s (399.8 eV), and O 1s (531.8 eV), confirming successful incorporation of N and O into the carbon framework [30]. The atomic percentages of C, N and O for three PCDs obtained from XPS full spectra are summarized in Table S1: *o*-PCDs (C 81.61%, N 1.73%, O 16.65%), *m*-PCDs (C 72.47%, N 7.28%, O 20.25%), *p*-PCDs (C 76.06%, N 8.02%, O 15.22%). The differences in elemental composition lead to distinct fluorescence responses to metal ions. The high-resolution C 1s spectrum (Figure S2b) reveals binding energies at 284.8 eV (C=C/C-C) and 285.9 eV (C-O/C-N). The N 1s spectrum (Figure S2c) shows peaks at 399.6 eV (N-H) and 401.0 eV (pyridinic N). Deconvolution of the O 1s spectrum (Figure S2d) yields peaks at 532.2 eV (C=O) and 533.8 eV (C-O) [31,32]. These results indicate abundant hydrophilic functional groups on the *o*-PCDs surface, consistent with FT-IR data. Similar spectral features were observed for *m*-PCDs and *p*-PCDs (Figures S3 and S4), verifying successful synthesis of all three PCDs variants. Variations in elemental content (Table S1) accounted for their differential responses to metal ions.

### 3.2. Optical Characteristics

**Figure 2:**
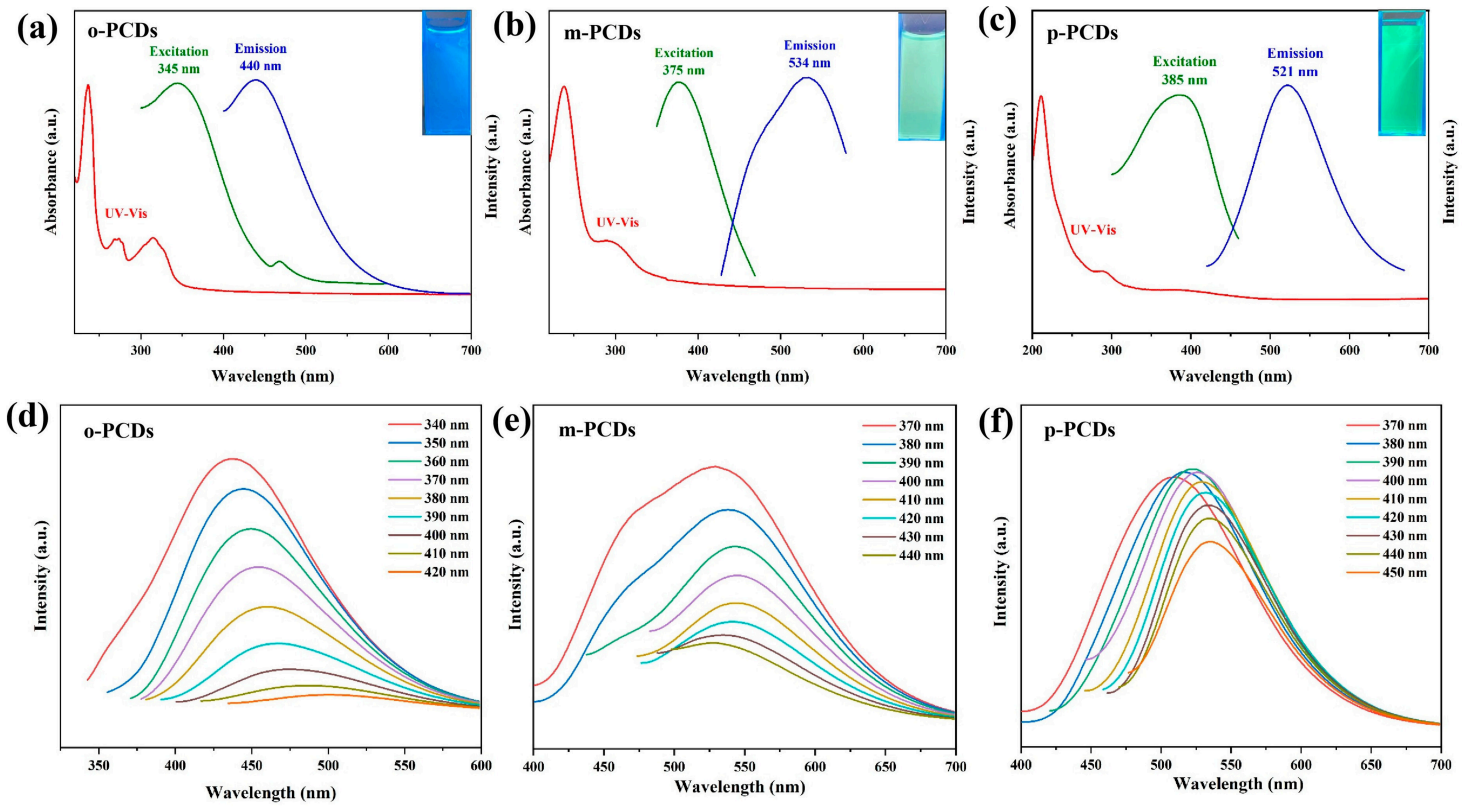
Optical characterization curves (inset: the aqueous solutions under 365 nm excitation) and fluorescence spectra under different excitation wavelengths of (**a**,**d**) *o*-PCDs; (**b**,**e**) *m*-PCDs; (**c**,**f**) *p*-PCDs.

The optical properties of *o*-PCDs, *m*-PCDs, and *p*-PCDs were evaluated through UV-vis absorption and fluorescence spectra. Figure 2a–c reveals a strong absorption peak at ∼230 nm corresponding to π-π* transition of C=C bonds, and a broad peak near 300 nm from n-π* transitions in C=N/C=O groups. *o*-PCDs exhibit an additional characteristic peak at 274 nm, assigned to π-π* transitions in aromatic sp^2^ domains [33,34,35]. Optimal excitation/emission wavelengths occur at 345/440 nm (o-PCDs), 375/534 nm (m-PCDs), and 385/521 nm (p-PCDs). The aqueous solutions emit blue, yellowish-green, and green fluorescence under 365 nm excitation, respectively (insets, Figure 2a–c). The fluorescence quantum yields (QYs) of three PCDs were measured using quinine sulfate as reference: *o*-PCDs (8.26%), *m*-PCDs (7.26%), *p*-PCDs (10.57%).

Fluorescence spectra (Figure 2d–f) demonstrate a progressive decrease in intensity and significant emission red-shifts for all PCDs as excitation wavelengths exceed their optima. The excitation-dependent behavior aligns with reported carbon dots and arises from core structural heterogeneity and surface defects [18].

In practical applications, the fluorescence stability of PCDs is significantly influenced by complex environmental conditions. This study investigated the effects of salt concentration (NaCl, 0–1.0 M) and various anions (H_2_PO_4_^−^, HPO_4_^2−^, CO_3_^2−^, F^−^, SO_4_^2−^, NO_3_^−^, and Cl^−^, each at 400 μM) on the fluorescence intensity of *o*-PCDs, *m*-PCDs, and *p*-PCDs. The results demonstrated that all three PCDs exhibited remarkable fluorescence stability even at NaCl concentrations up to 1.0 M (Figure S5a). Notably, H_2_PO_4_^−^, HPO_4_^2−^, and CO_3_^2−^ induced significant fluorescence changes, attributable to anion-induced pH alterations. In contrast, F^−^, SO_4_^2−^, NO_3_^−^, and Cl^−^ exhibited negligible effects, confirming that these anions do not interfere with the detection process (Figure S5b).

### 3.3. Construction of the Sensor Array

#### 3.3.1. Fluorescence Response of Metal Ions to the PCDs

**Figure 3:**
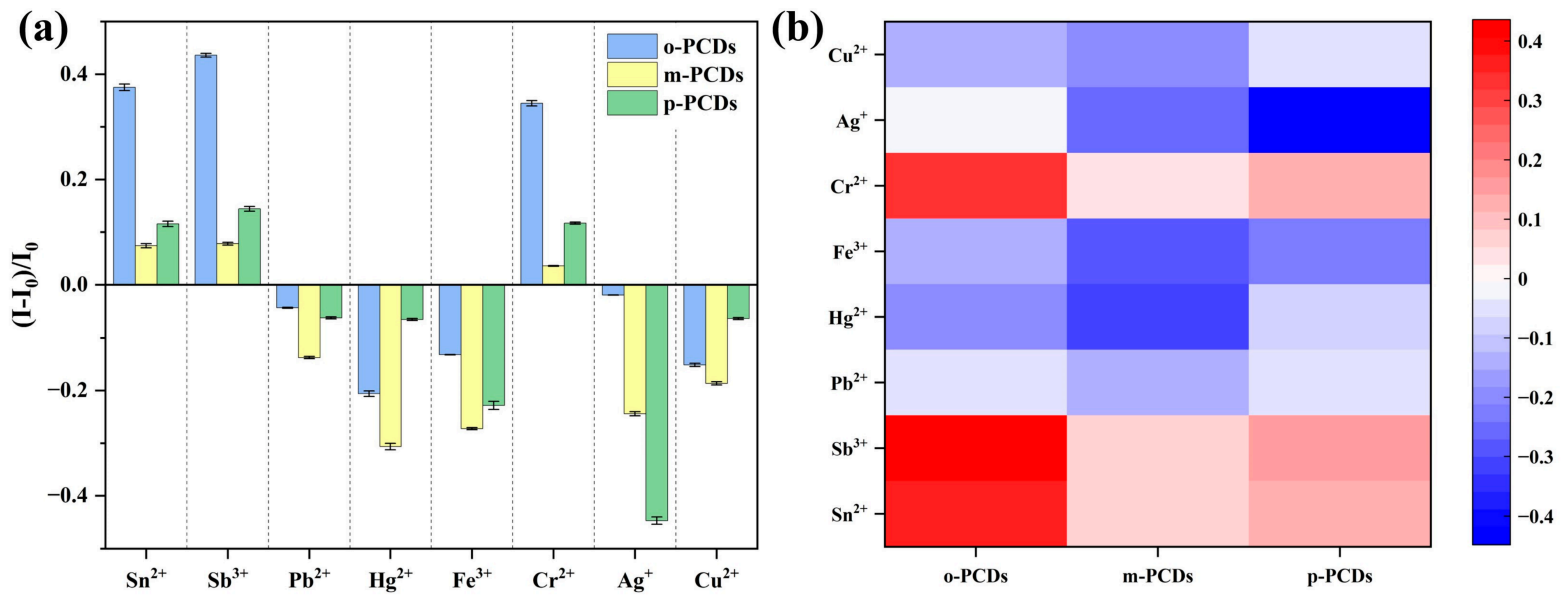
(**a**) The fluorescent response profiles and (**b**) heat map of eight metal ions (400 μM).

The fluorescence intensity was measured before and after the addition of metal ions, and the response was quantified as (I − I_0_)/I_0_. The fluorescence responses of *o*-PCDs, *m*-PCDs, and *p*-PCDs to Sn^2+^, Ag^+^, Hg^2+^, Fe^3+^, Cr^3+^, Pb^2+^, Sb^3+^, and Cu^2+^ (each at 400 μM) were investigated. These eight ions are typical toxic heavy metals widely monitored in surface water and industrial wastewater. The distinctive fluorescence profiles of these metal ions are revealed (Figure 3a), and their differences allow for clear differentiation in the corresponding heatmap (Figure 3b). Concentration-dependent studies (5–400 μM) showed significant variations in fluorescence enhancement or quenching among different ions (Figure S6). Specifically, *o*-PCDs exhibited the highest selectivity for Sb^3+^ with 43.6% enhancement, *m*-PCDs showed the strongest quenching toward Hg^2+^ (30.6%), and *p*-PCDs displayed optimal quenching for Ag^+^ (44.7%).

These differential responses arise from the intrinsic properties of the PCDs and the metal ions. Distinct precursors lead to differences in particle size, elemental composition, and surface functional groups among the three PCDs, as evidenced by TEM, FT-IR, UV–vis, XPS, and fluorescence spectra. Furthermore, the orbital energy levels, electronic properties, and chelating capacity of the metal ions with surface functional groups collectively determine the specificity of fluorescence quenching or enhancement [24]. Therefore, a sensing array based on *o*-PCDs, *m*-PCDs, and *p*-PCDs can utilize their distinct fluorescence response patterns for efficient discrimination and detection of metal ions.

#### 3.3.2. Discrimination of the Sensor Array to Metal Ions with Varying Concentrations

A fluorescent sensor array was fabricated using *o*-PCDs, *m*-PCDs, and *p*-PCDs as sensing elements. The fluorescence response, defined as (I − I_0_)/I_0_, was quantified for the eight metal ions (Sn^2+^, Ag^+^, Hg^2+^, Fe^3+^, Cr^3+^, Pb^2+^, Sb^3+^, and Cu^2+^) at a concentration of 400 μM, with five replicates per ion. This yielded a three-dimensional dataset structured as 3 (sensors) × 8 (ions) × 5 (repeats).

**Figure 4:**
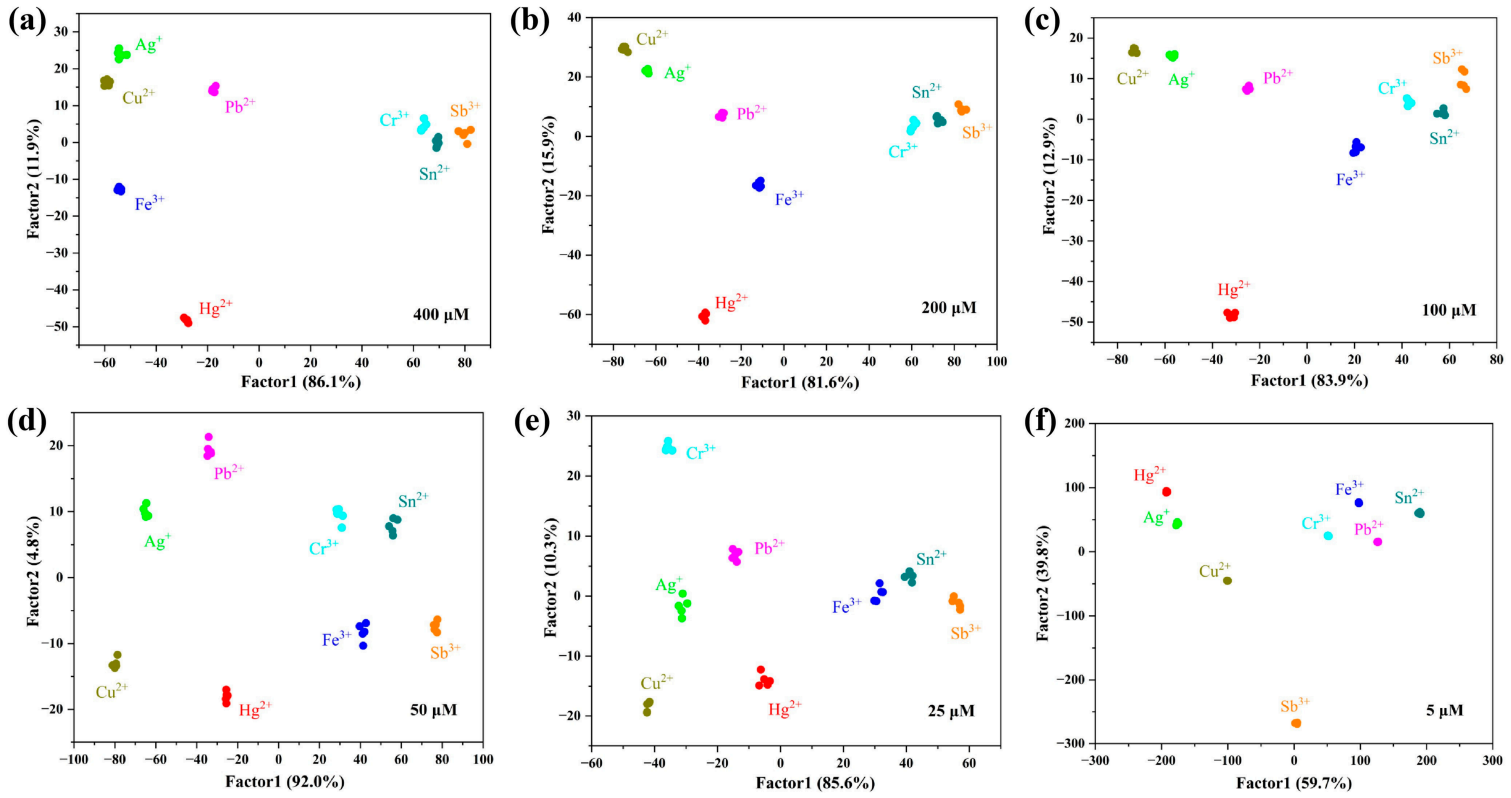
LDA canonical score plot of the array to eight heavy metal ions at (**a**) 400, (**b**) 200, (**c**) 100, (**d**) 50, (**e**) 25, and (**f**) 5 μM under 365 nm excitation.

Linear discriminant analysis (LDA) was applied for supervised pattern recognition to reduce dimensionality while maximizing inter-class separation and minimizing intra-class variance [36]. As shown in Figure 4a, the data were projected into two discriminant factors, accounting for 86.1% (Factor 1) and 11.9% (Factor 2) of the total variance. All eight metal ions formed distinct and well-separated clusters in the discriminant space with no observed misclassification. The five replicates clustered tightly, demonstrating excellent reproducibility and stability of the array [37]. Cross-validation and confusion matrix results further confirm that the model has no obvious overfitting, and the discrimination performance is reliable.

**Figure 5:**
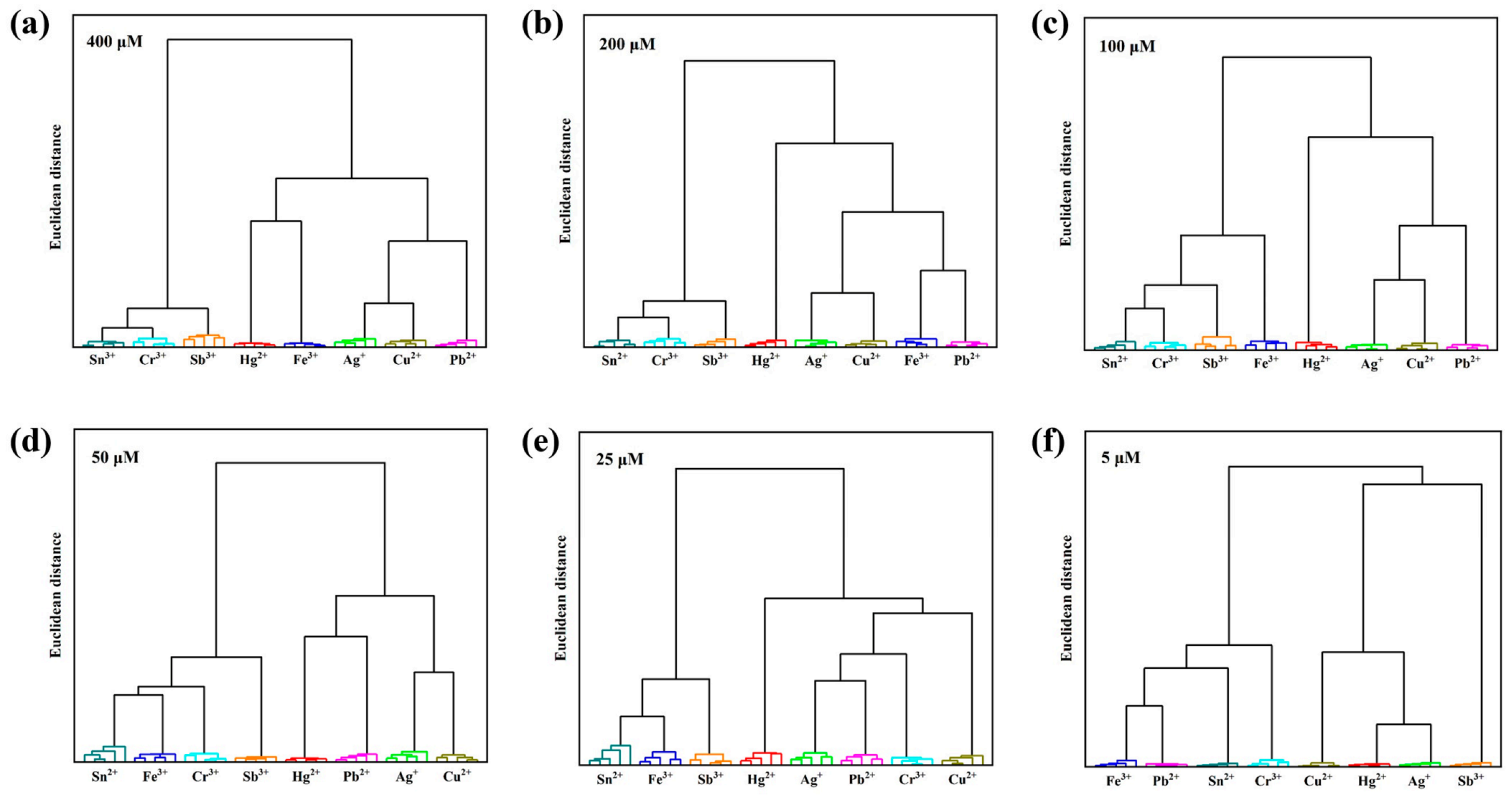
HCA tree graph of the array to eight metal ions at (**a**) 400, (**b**) 200, (**c**) 100, (**d**) 50, (**e**) 25, and (**f**) 5 μM under 365 nm excitation.

Hierarchical clustering analysis (HCA) further validated the discrimination capability, yielding dendrograms with 100% classification accuracy across all ions (Figure 5a). Moreover, the array consistently differentiated all target ions at a range of concentrations (5–200 μM), with a lowest detectable concentration of 5 μM (Figure 4b–f and Figure 5b–f). These results highlight the potential of the sensor array for practical use in trace metal ion identification [38]. The performance of this array was quantitatively compared with recently reported CD-based sensing arrays in Table S2, including detection range, response time, detectable ion species and anti-interference performance. This array has a wide detection range, fast response (1 min) and excellent cross-selectivity.

#### 3.3.3. Effect of pH on Discrimination

**Figure 6:**
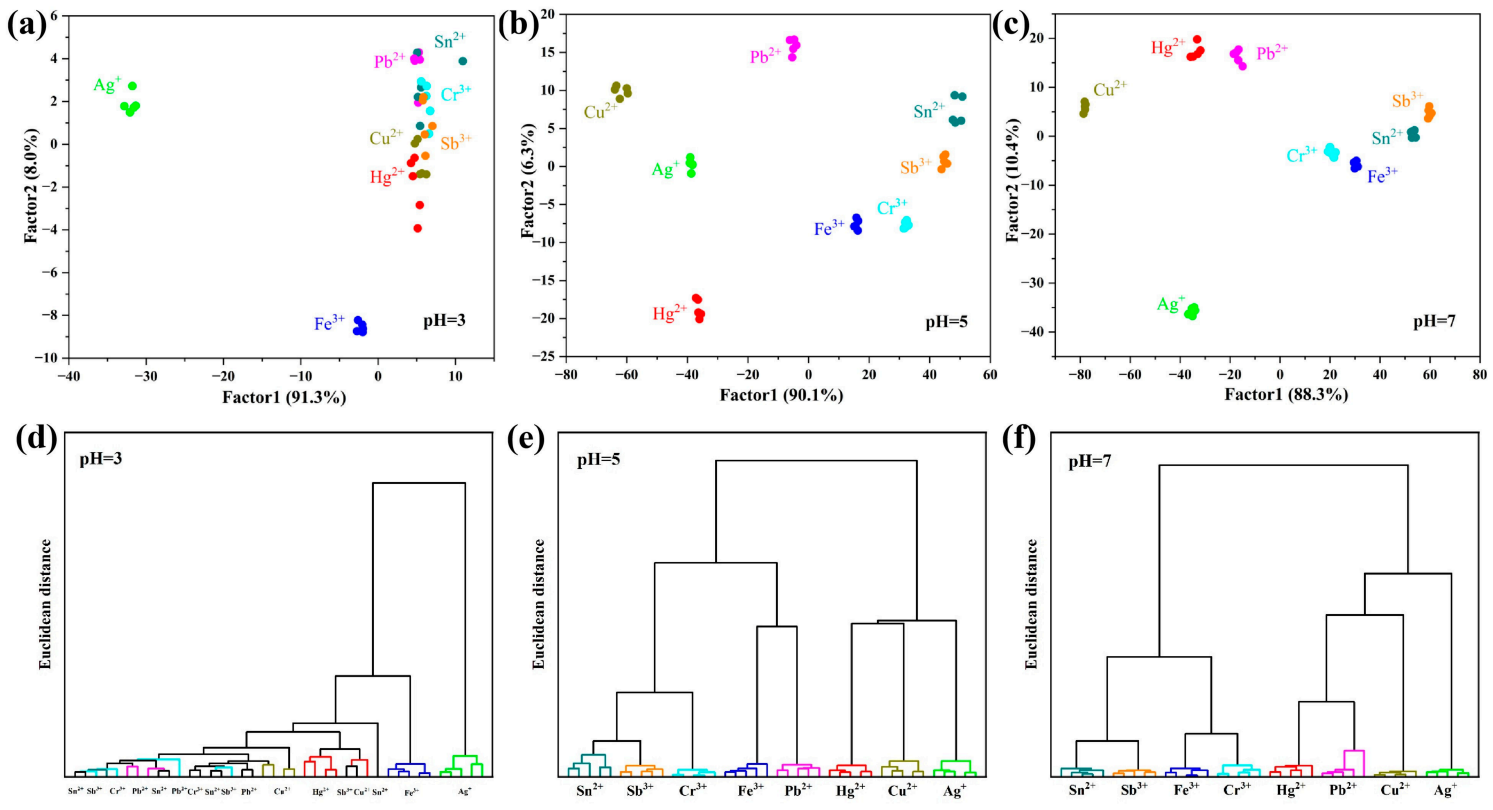
LDA canonical score plot and HCA tree graph for eight heavy metal ions analyzed with the sensor array at (**a**,**d**) pH = 3, (**b**,**e**) pH = 5, and (**c**,**f**) pH = 7 under 365 nm excitation.

Determining the optimal pH range is critical for the metal-ion detection performance of the sensor array. Since many metal ions form hydroxide precipitates in alkaline media [39], the pH range from 3 to 7 was selected for evaluation. LDA (Figure 6a–c) and HCA (Figure 6d–f) showed that at pH 3, only Fe^3+^ and Ag^+^ were clearly distinguishable, while Sn^2+^, Hg^2+^, Cr^3+^, Pb^2+^, Sb^3+^, and Cu^2+^ exhibited substantial clustering overlap and misclassification, precluding reliable identification. In contrast, the pH range of 5–7 was optimal, enabling clear discrimination of all target ions. These findings suggest that the PCDs maintain a stable conjugated structure under neutral to weakly acidic conditions (pH 5–7), facilitating distinct responses to different metal ions. At lower pH (e.g., pH 3), protonation-induced structural disruption likely compromises conjugation, resulting in dominant fluorescence interference from H^+^ over metal ion signals and suppression of characteristic response signals [24].

### 3.4. Discrimination of Binary and Ternary Metal Ion Mixtures

**Figure 7:**
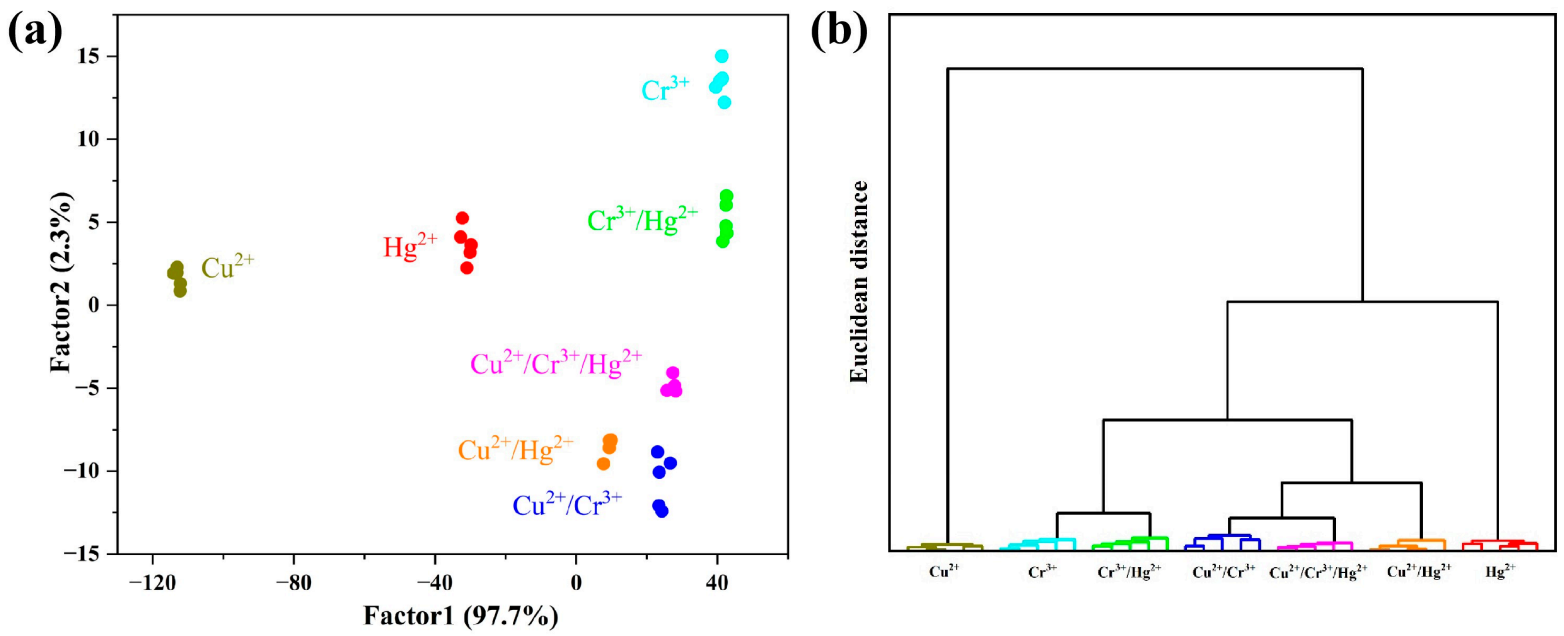
(**a**) LDA canonical score plot and (**b**) HCA tree graph for metal ion mixtures analyzed with the sensor array.

To evaluate the discrimination capability of the sensing array in a multi-ion system, HCA and LDA were employed to analyze four metal ion mixtures (Cu^2+^/Hg^2+^, Cu^2+^/Cr^3+^, Cr^3+^/Hg^2+^, and Hg^2+^/Cr^3+^/Cu^2+^), each with a 1:1 molar ratio and a total concentration of 100 μM. The LDA score plot differentiated all mixtures with 100% accuracy, and replicates of the same mixture formed tight clusters (Figure 7a). This result was further confirmed by the HCA dendrogram (Figure 7b), in which the seven distinct mixture groups were accurately clustered and ultimately converge. These results demonstrate that the fluorescence sensor array based on *o*-PCDs, *m*-PCDs, and *p*-PCDs exhibits significant potential for the identification of complex metal ion mixtures.

### 3.5. Fluorescence Regulation Mechanisms of the PCDs

In fluorescent sensor arrays, the response of metal ions to PCDs may originate from intermolecular interactions, such as weak metal–metal interactions, π–π stacking, hydrogen bonding, and C–H···π interactions, triggered by incorporation into the PCDs lattice. Additionally, coordination between metal ions and oxygen/nitrogen-containing functional groups (e.g., hydroxyl, amino, and carboxyl groups) contributes to the fluorescence changes [40].

To elucidate the fluorescence response mechanisms, the most responsive metal ions for *o*-PCDs, *m*-PCDs, and *p*-PCDs were investigated. Fluorescence lifetimes were measured before and after addition of each ion (200 μM), and UV-vis spectra were recorded for PCDs incubated with metal ions (5–400 μM).

In the presence of Sb^3+^, the average fluorescence lifetime of *o*-PCDs increased significantly from 5.62 ns to 7.05 ns (Figure S7a), with no shift or new peaks in the UV-vis spectrum (Figure S8a). Due to the combination of multiple quenching modes, the Stern–Volmer curves at different temperatures exhibit a downward shift with increasing temperature, consistent with static quenching characteristics (Figure S9a). According to Zeta potential measurements, upon addition of Sb^3+^, the potential of *o*-PCDs gradually becomes more positive, with the Zeta potential rising from −8 mV to −3.6 mV (Figure S9b) [41,42], which strongly suggests that a chelating complex, formed via the coordination of Sb^3+^ with O and N atoms, enhanced the fluorescence efficiency [43,44]. Upon addition of Hg^2+^, the lifetime of *m*-PCDs remained stable (4.15 ns → 4.08 ns, Figure S7b), while the absorption spectrum exhibited a blue shift with increasing Hg^2+^ concentration (Figure S8b). At different temperatures, the quenching constant Stern–Volmer curve continuously decreases with increasing temperature (Figure S9c), exhibiting characteristics consistent with static quenching; simultaneously, static quenching alters the surface potential. In *m*-PCDs solutions, upon addition of Hg^2+^ solution, the Zeta potential decreases from +20.4 mV to +2.8 mV (Figure S9d) [45,46,47], which is consistent with confirming the formation of a ground-state complex consistent with static quenching [48]. After addition of Ag^+^, the average lifetime of *p*-PCDs decreased from 6.92 ns to 6.20 ns (Figure S7c), with no change in their absorption spectra (Figure S8c), indicating a possible dynamic quenching through collisional deactivation of the excited state [49]. The above data provide auxiliary evidence for mechanism inference, and more in-depth theoretical and experimental research will be carried out in follow-up work. At different temperatures, the quenching constant Stern–Volmer curve increases with rising temperature (Figure S9e). Furthermore, upon addition of an Ag+ solution, the Zeta potential of the *p*-PCDs solution rises from +14.2 mV to +15 mV (Figure S9f), while the surface charge shows no significant change, all consistent with dynamic quenching characteristics [50].

### 3.6. Application of the Sensor Array in Real Samples

To assess the identification performance of the sensor array for metal ions in environmental water samples, standard solutions of Sn^2+^, Ag^+^, Hg^2+^, Fe^3+^, Cr^3+^, Pb^2+^, Sb^3+^, and Cu^2+^ at 100 μM were added into environmental water matrices. LDA showed complete separation of the target ions into eight distinct clusters with 100% classification accuracy (Figure S9a). This result was corroborated by HCA, where all ions formed independent clustering branches (Figure S9b). These findings confirm the excellent stability and applicability of the fluorescence sensor array for accurate metal ion identification in environmental water samples.

Compared to existing sensor arrays (Table S2), this work presents distinct advantages. Different from previously reported CD-based arrays, this work uses novel black plum peel biomass waste as carbon source and three phenylenediamine isomers to construct a three-channel array. The raw materials are green and low-cost, and the synthesis requires no post-modification. The array employs biomass waste-derived PCDs, which are simple to synthesize and require no post-modification. Using only three sensing units, the array enables high-throughput discrimination of eight metal ions, offering a broad detection range, short response time, and successful real-sample application. This approach provides an efficient strategy for rapid metal ion screening and extends the applicability of fluorescent sensor arrays.

## 4. Conclusions

This work successfully constructed a novel fluorescent sensor array using agricultural waste black plum peel as a carbon source and phenylenediamine isomers as nitrogen dopants. The PCDs are synthesized via a one-step hydrothermal method with simple operation, low cost and easy large-scale production, showing good economic feasibility and application potential. The array can effectively discriminate eight typical toxic heavy metal ions in the concentration range of 5–400 μM under pH 5–7, with fast response and excellent anti-interference ability. Combined with LDA and HCA, it achieves reliable identification of single ions, mixed ions and actual river water samples.

The main limitations of this work are as follows: only eight heavy metal ions were selected for detection, and the performance for ultra-trace heavy metal ions needs further optimization; the current application is limited to environmental water monitoring, and biological sample detection has not been explored.

In future research, we will expand the range of detectable metal ions and further improve the limit of detection for ultra-trace heavy metal pollutants. Benefiting from the excellent biocompatibility of these PCDs, we will carry out fetal bovine serum matrix spike recovery experiments and intracellular heavy metal ion fluorescence imaging to systematically explore biological sensing performance. We also plan to develop portable integrated testing devices for on-site environmental screening. This work offers a promising strategy for qualitative heavy metal identification in environmental monitoring.

## Data Availability

Data is contained within the article or supplementary material.

## Supplementary Materials

The following supporting information can be downloaded at: https://www.mdpi.com/article/10.3390/bios16070372/s1, Figure S1: FT-IR spectra of *o*-PCDs, *m*-PCDs, and *p*-PCDs; Figure S2: XPS spectra of *o*-PCDs; Figure S3: XPS spectra of *m*-PCDs; Figure S4: XPS spectra of *p*-PCDs; Figure S5: Effects of NaCl concentration and various anions on the fluorescence intensity of *o*-PCDs, *m*-PCDs and *p*-PCDs; Figure S6: Concentration-dependent fluorescence responses of *o*-PCDs, *m*-PCDs, and *p*-PCDs to eight metal ions upon excitation at 365 nm; Figure S7: Fluorescence lifetime of *o*-PCDs and *o*-PCDs + Sb^3+^; *m*-PCDs and *m*-PCDs + Hg^2+^, *p*-PCDs and *p*-PCDs + Ag^+^; Figure S8: UV-vis spectra of *o*-PCDs, *m*-PCDs, and *p*-PCDs in the absence and presence of metal ions at different concentrations; Figure S9: Temperature-dependent Stern-Volmer plots of *o*-PCDs with Sb^3^^+^, Zeta potentials of *o*-PCDs and *o*-PCDs + Sb^3^^+^, Temperature-dependent Stern-Volmer plots of *m*-PCDs with Hg^2^^+^, Zeta potentials of *m*-PCDs and *m*-PCDs + Hg^2^^+^, Temperature-dependent Stern-Volmer plots of *p*-PCDs with Ag^+^, Zeta potentials of *p*-PCDs and *p*-PCDs + Ag^+^; Figure S10: LDA canonical score plot and HCA tree graph of the sensor array to eight heavy metal ions with environmental water at 100 μM; Table S1: Element Contents of XPS analysis; Table S2: Comparison of different methods for the discrimination of metal ions. References [51,52,53,54,55,56] are cited in the supplementary materials.
