# Supplementary material for: Fluorescent Sensor Array Based on Black Plum Peels-Derived Carbon Dots for Multiplex Heavy Metal Ions Identification
Source: Biosensors (Basel). 2026 Jul 8;16(7):372. doi: 10.3390/bios16070372 (PMC13406801; doi:10.3390/bios16070372)
Supplement: Supplementary file 1 [file biosensors-16-00372-s001.zip › biosensors-4309198-supplementary.pdf]

Electronic Supplementary material

Fluorescent Sensor Array Based on Black Plum Peels-Derived Carbon Dots for Multiplex Heavy Metal Ions Identification

Ling Yang [1,2], Dandan Peng [1], Haihu Tan [1,*], Yahu Wang [1], Xin Lu [1], Fanming Zeng [3], Shigang Liu [4] and Yuejun Liu [2,*]

[1] School of Packaging Engineering, Hunan University of Technology, Zhuzhou 412007, China; yangling2020@hut.edu.cn (L.Y.)
[2] National & Local Joint Engineering Research Center for Advanced Packaging Material and Technology, Hunan University of Technology, Zhuzhou 412007, China
[3] College of Materials Science and Engineering, Fuzhou University, Fuzhou 350108, China
[4] Hunan Provincial Key Laboratory of Food Science and Biotechnology, College of Food Science and Technology, Hunan Agricultural University, Changsha 410128, China
* Correspondence: tanhaihu2020@hut.edu.cn (H.T.); yjliu_2005@126.com (Y.L.)

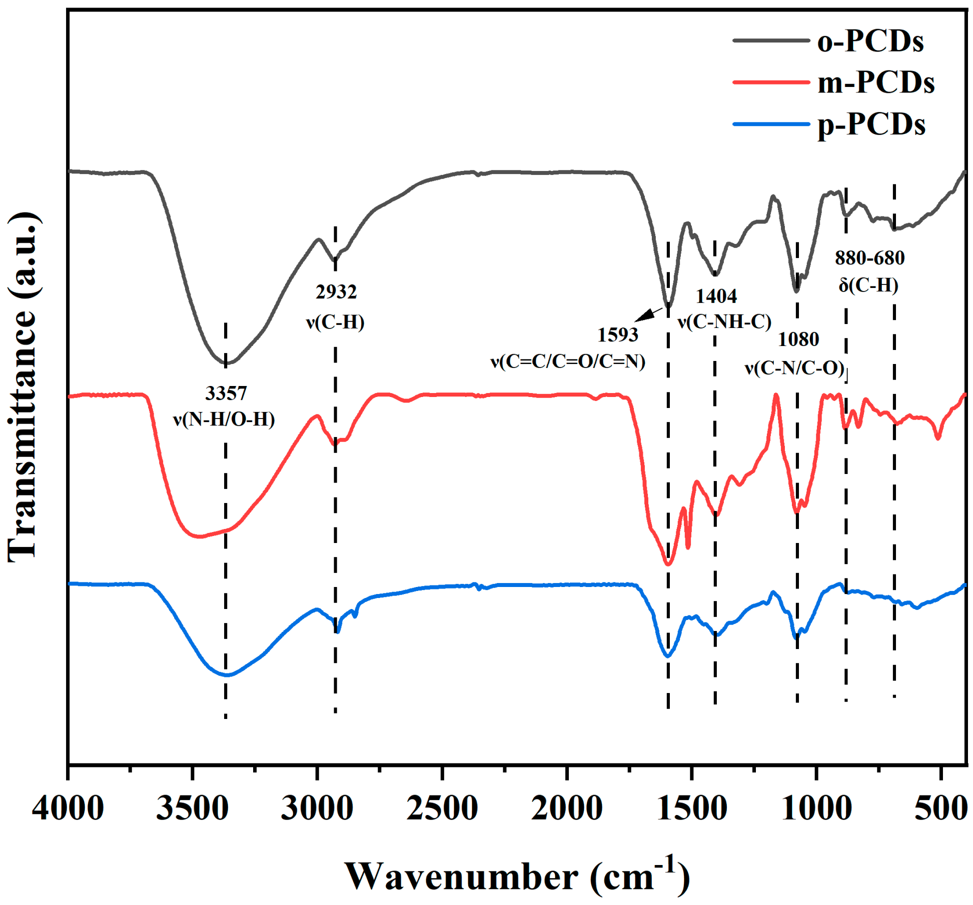

Fig. S1 FT-IR spectra of o-PCDs, m-PCDs, and p-PCDs.

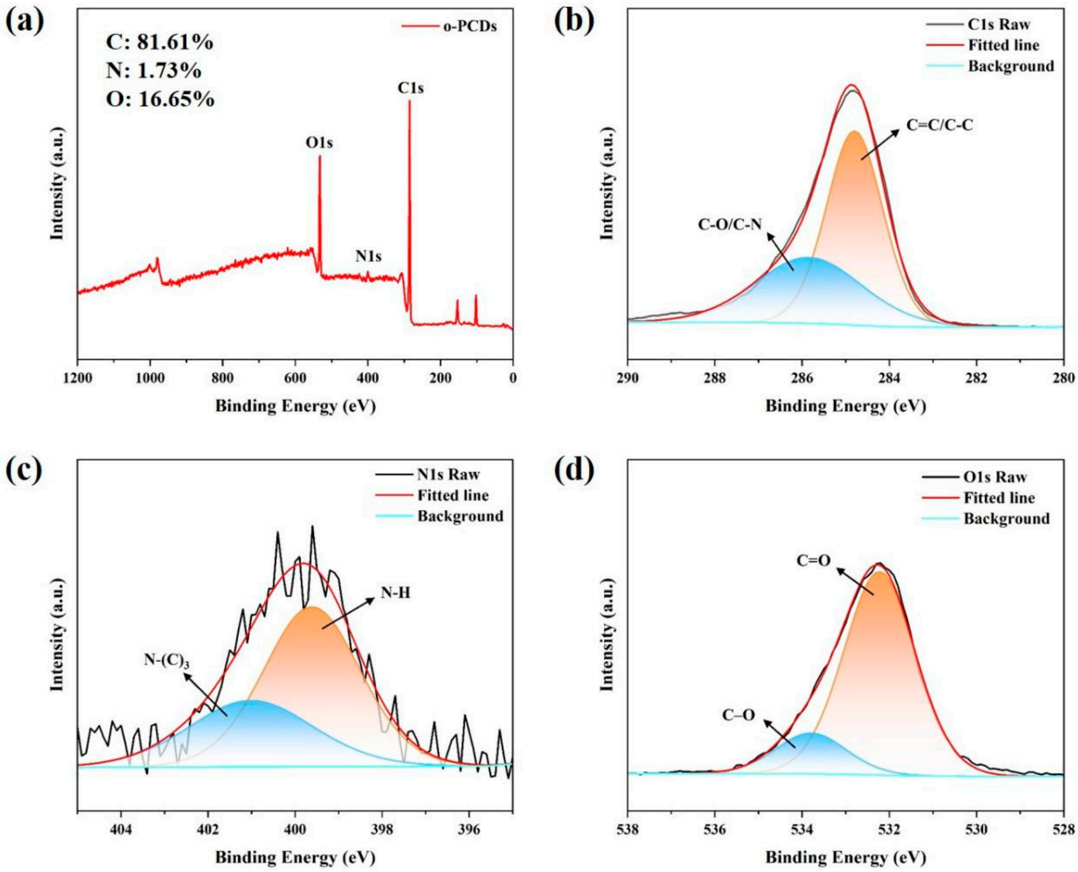

Fig. S2 XPS spectra of o-PCDs. (a) Full spectrum; (b) C1s fine spectrum; (c) N1s fine spectrum; (d) O1s fine spectrum.

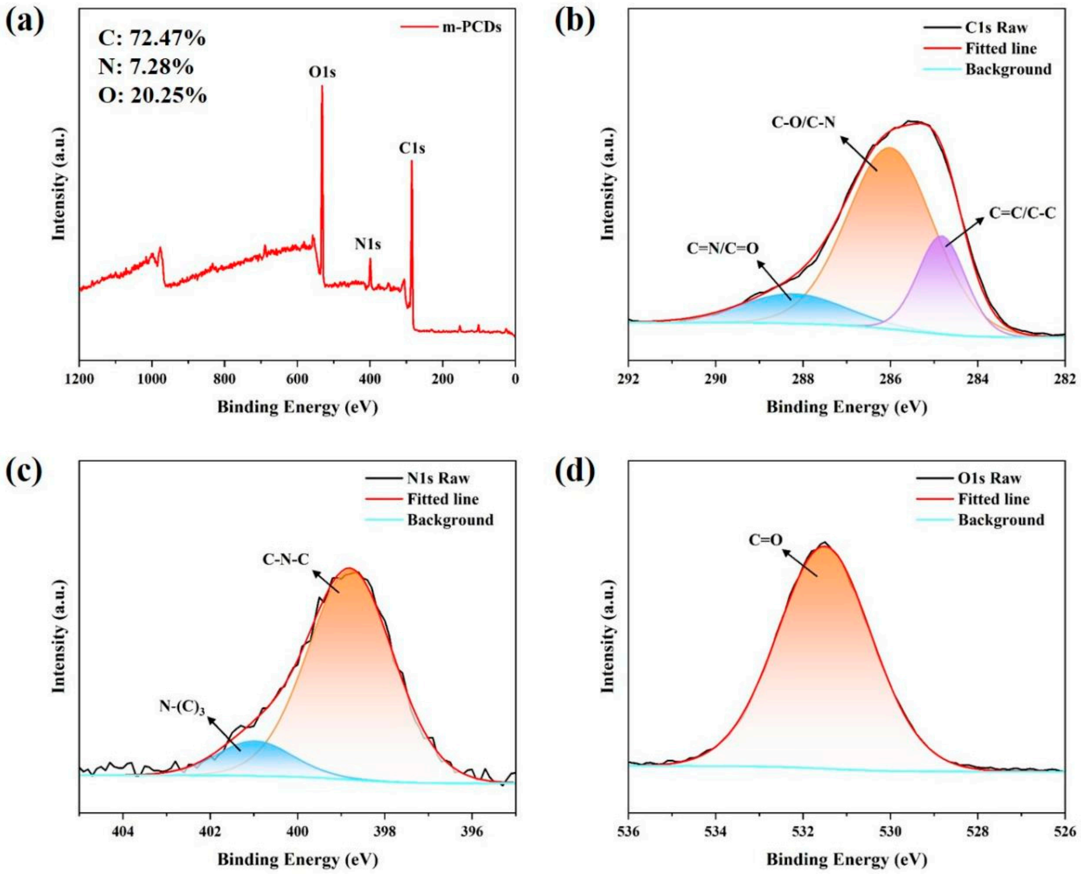

Fig. S3 XPS spectra of m-PCDs. (a) Full spectrum; (b) C1s fine spectrum; (c) N1s fine spectrum; (d) O1s fine spectrum.

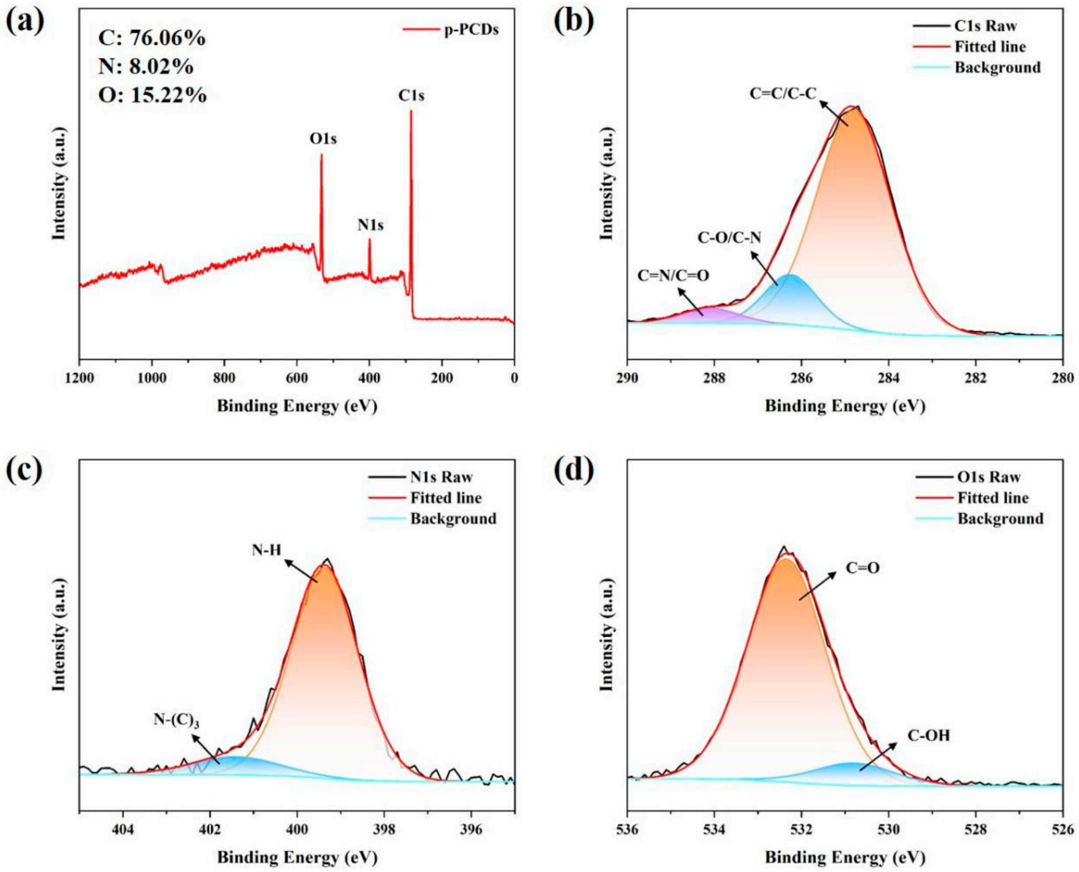

Fig. S4 XPS spectra of p-PCDs. (a) Full spectrum; (b) C1s fine spectrum; (c) N1s fine spectrum; (d) O1s fine spectrum.

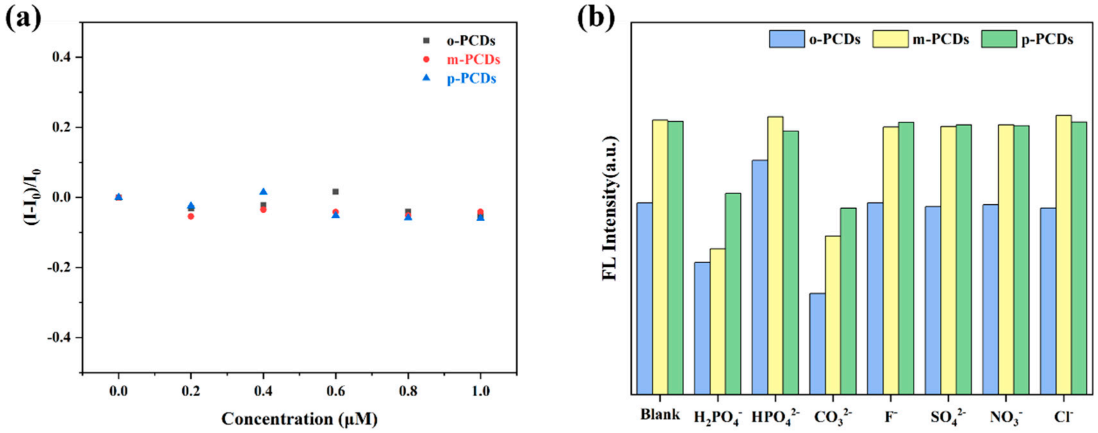

Fig. S5 Effects of (a) NaCl concentration (0-1.0 M) and (b) various anions ($H_2PO_4^-$, HPO_4^{2-}, CO_3^{2-}, F^-, SO_4^{2-}, NO_3^-, and Cl^-, each at 400 μM) on the fluorescence intensity of o-PCDs, m-PCDs and p-PCDs.

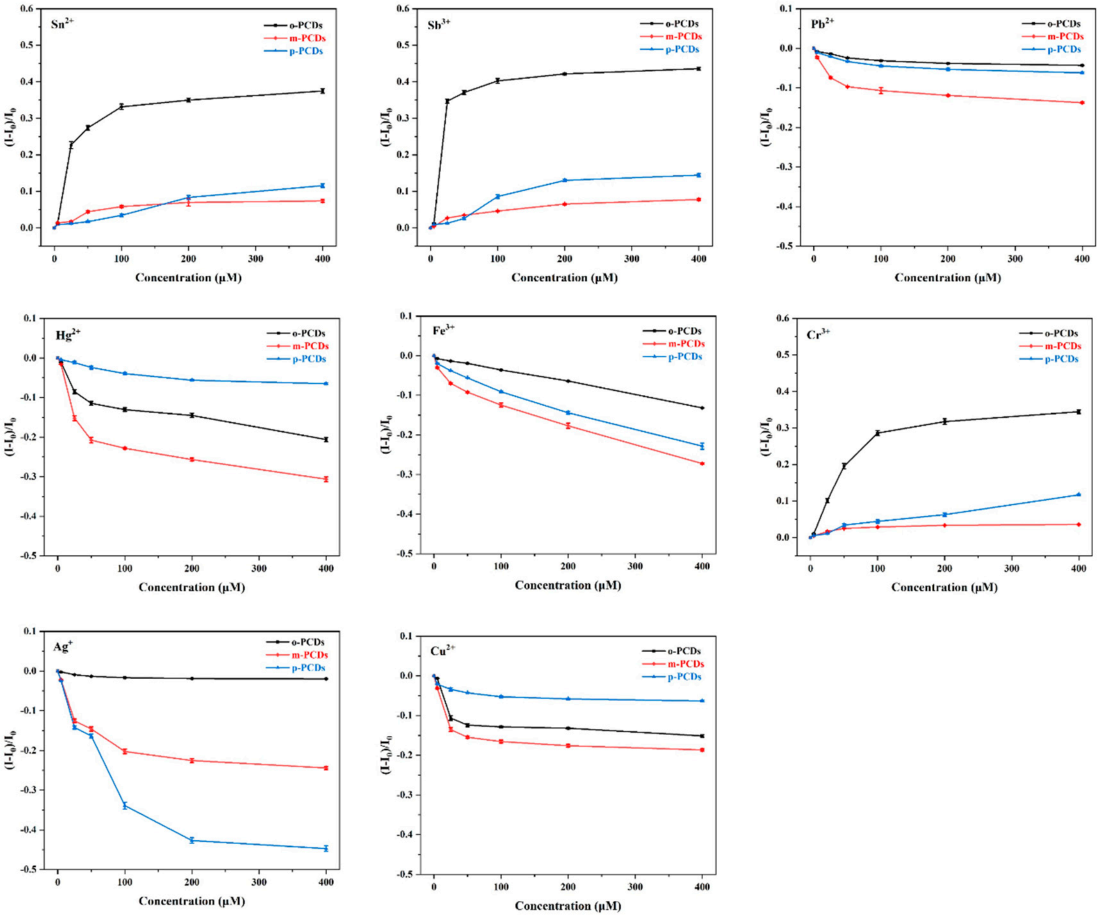

Fig. S6 Concentration-dependent fluorescence responses of o-PCDs, m-PCDs, and p-PCDs to eight metal ions upon excitation at 365 nm.

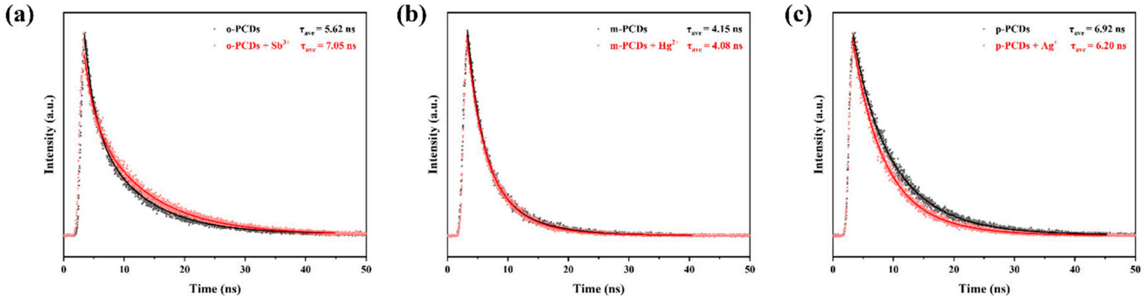

Fig. S7 Fluorescence lifetime of (a) o-PCDs and o-PCDs + Sb^{3+}; (b) m-PCDs and m-PCDs + Hg^{2+}, (c) p-PCDs and p-PCDs + Ag^{+}.

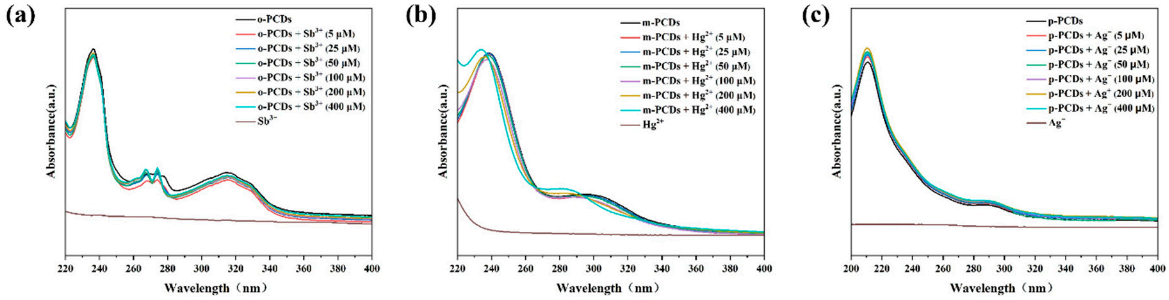

Fig. S8 UV-vis spectra of (a) o-PCDs, (b) m-PCDs, and (c) p-PCDs in the absence and presence of metal ions at different concentrations (5-400 μM).

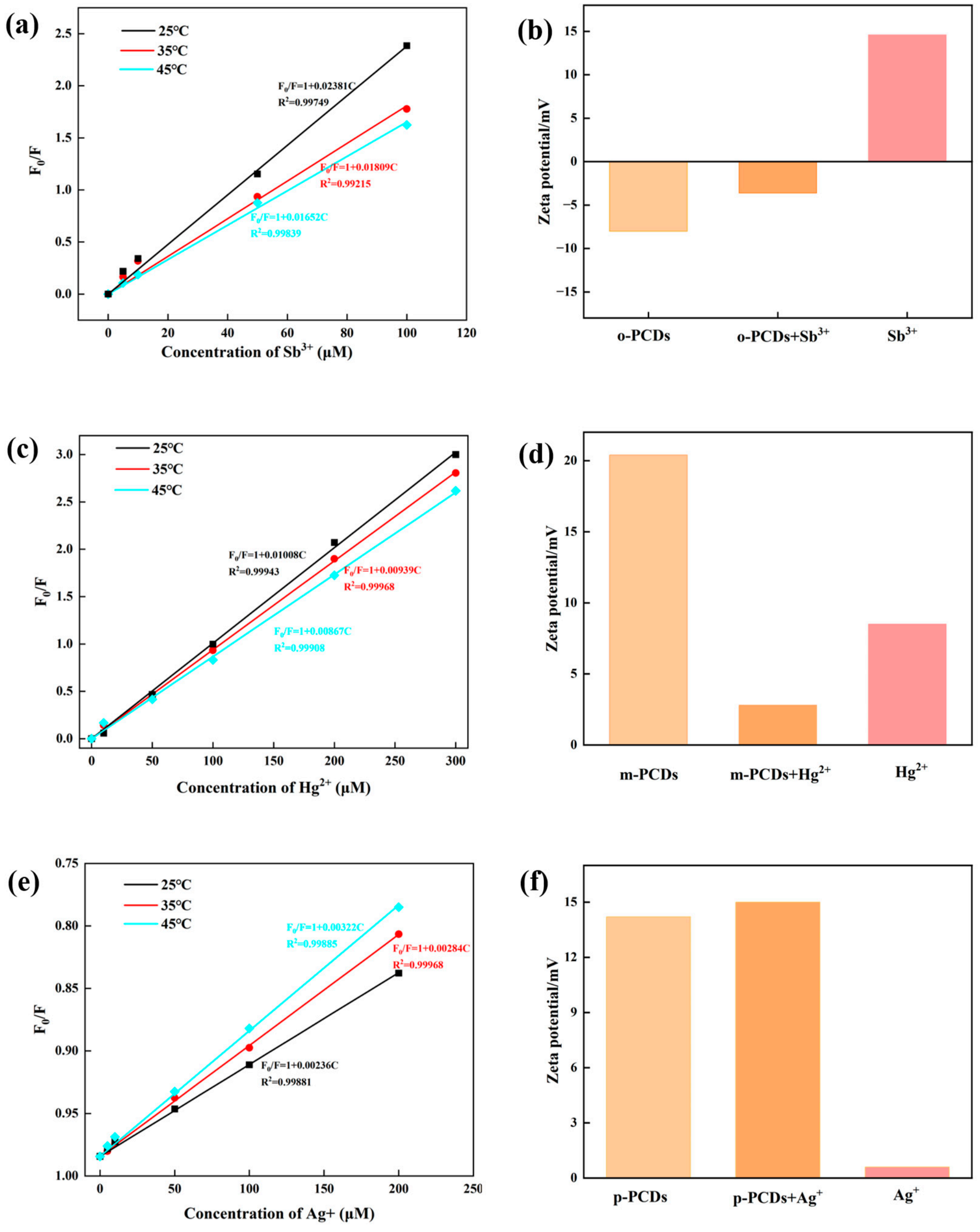

Fig. S9 (a) Temperature-dependent Stern-Volmer plots of o-PCDs with Sb^{3+}; (b) Zeta potentials of o-PCDs and o-PCDs + Sb^{3+}; (c) Temperature-dependent Stern-Volmer plots of m-PCDs with Hg^{2+}; (d) Zeta potentials of m-PCDs and m-PCDs + Hg^{2+}; (e) Temperature-dependent Stern-Volmer plots of p-PCDs with Ag^{+}; (f) Zeta potentials of p-PCDs and p-PCDs + Ag^{+}.

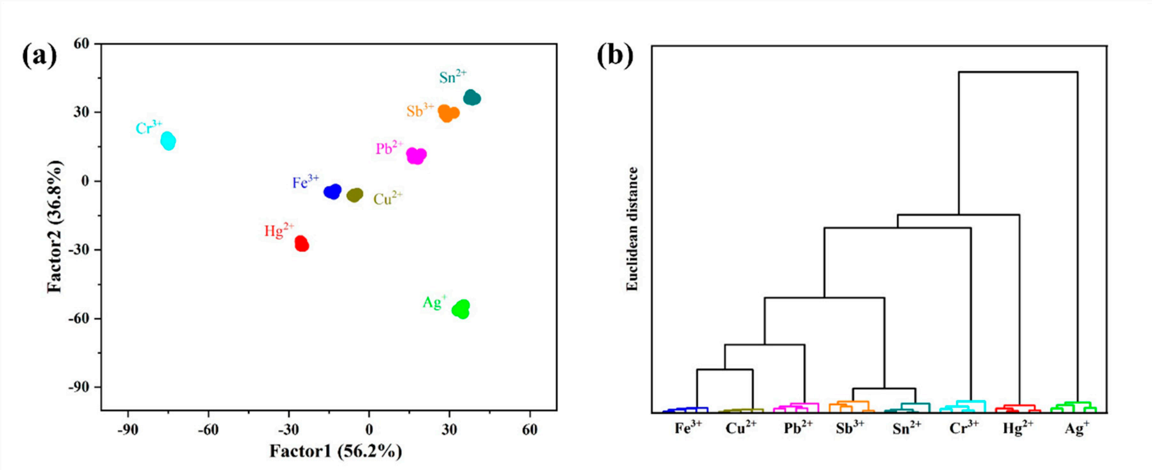

Fig. S10 (a) LDA canonical score plot and (b) HCA tree graph of the sensor array to eight heavy metal ions with environmental water at 100 μM.

Tab. S1 Element Contents of XPS analysis.

Element	o-PCDs	m-PCDs	p-PCDs
C 1s (%)	81.61	72.47	76.06
N 1s (%)	1.73	7.28	8.02
O 1s (%)	16.65	20.25	15.22

Tab. S2 Comparison of different methods for the discrimination of metal ions.

**Sensors**	**Detection method**	**Number of sensing units**	**Detection range**	**Metal ions**	**Response time (min)**	**Samples**	**Ref**
AC-CQDs functionalized with different amino acids	FL spectrophotometer (array)	6	5-100 μM	Mo^{5+}, Fe^{3+}, Cr^{3+}, Er^{3+}, Yb^{3+}, La^{3+}, Ni^{2+}, Cu^{2+}, Co^{2+}, Pb^{2+} and Mn^{2+}	30	Tap water and river water	[3]
Biomass CDs	FL spectrophotometer (array)	3	0.04-4 μM	Ag^{+}, Cu^{2+}, Hg^{2+}, Fe^{3+} and Pb^{2+}	5	River water	[24]
Tri-emission QR-CDs / EDTA-Tb^{3+}	FL spectrophotometer (array)	1	0.05-50 μM	Cr^{6+}, Fe^{2+}, Cu^{2+}, Fe^{3+}, Mn^{2+}, Co^{2+} and Ni^{2+}	/	Lake water and soil	[25]
Biomass CDs	FL spectrophotometer (array)	3	5-100 μM	Cu^{2+}, Fe^{3+}, Mn^{2+}, Ag^{+}, Ce^{4+} and Ni^{2+}	1	Lake water	[31]
Tri-emission carbon dots (T-CDs)	FL spectrophotometer (array)	1	30-330 μM	Cd^{2+}, Co^{2+}, Cu^{2+}, Fe^{3+}, Mn^{2+} and Zn^{2+}	/	Tap water	[51]
Gallate-modified polymer dots (Pdots)	FL spectrophotometer (array)	4	0.5-50 μg/mL	Al^{3+}, Cu^{2+}, Co^{2+}, Ni^{2+}, Mg^{2+}, Pb^{2+}, Ba^{2+} and Ca^{2+}	/	Packaged water	[52]
N-CDs	FL spectrophotometer (array)	3	10-100 μM	Cr^{3+}, Cu^{2+}, Eu^{3+}, Fe^{3+}, Hg^{2+} and Pb^{2+}	/	/	[53]
Biomass CDs	Colorimetric sensor array	1	10-100 μM	Fe^{2+}, Fe^{3+}, Pb^{2+} and Hg^{2+}	/	Deionized water and spring water	[54]
NS-CDs	FL spectrophotometer (array)	3	5 – 500 μM/10-500 μM	Cr^{6+},Hg^{2+},and Mn^{7+}	1/3	Tap water	[55]
CR-CDs	FL spectrophotometer (array)	6	0.05 – 50 μM	Ag^{+}, Al^{3+}, Cd^{2+}, Co^{2+}, Cr^{6+}, Cr^{3+}, $Cu2^{+}$, Fe^{3+}, Fe^{2+}, Hg^{2+}, Mn^{2+}, Ni^{2+},	/	Tap water, Lake water, Yunnan red soil	[56]

				Pb^{2+}, Sn^{2+}, Ti^{4+}		leachate, coastal seawater, metal ion mixtures	
Biomass PCDs	FL spectrophotometer (array)	3	5-400 μM	Sn^{2+}, Ag^{+}, Hg^{2+}, Fe^{3+}, Cr^{3+}, Pb^{2+}, Sb^{3+}, and Cu^{2+}	1	River water	This work
